# IP-FCM Measures Physiologic Protein-Protein Interactions Modulated by Signal Transduction and Small-Molecule Drug Inhibition

**DOI:** 10.1371/journal.pone.0045722

**Published:** 2012-09-21

**Authors:** Stephen E. P. Smith, Anya T. Bida, Tessa R. Davis, Hugues Sicotte, Steven E. Patterson, Diana Gil, Adam G. Schrum

**Affiliations:** 1 Department of Immunology, Mayo Clinic College of Medicine, Rochester, Minnesota, United States of America; 2 Division of Biomedical Statistics and Informatics, Mayo Clinic, Rochester, Minnesota, United States of America; 3 Center for Drug Design, University of Minnesota, Minneapolis, Minnesota, United States of America; University Paris Sud, France

## Abstract

Protein-protein interactions (PPI) mediate the formation of intermolecular networks that control biological signaling. For this reason, PPIs are of outstanding interest in pharmacology, as they display high specificity and may represent a vast pool of potentially druggable targets. However, the study of physiologic PPIs can be limited by conventional assays that often have large sample requirements and relatively low sensitivity. Here, we build on a novel method, immunoprecipitation detected by flow cytometry (IP-FCM), to assess PPI modulation during either signal transduction or pharmacologic inhibition by two different classes of small-molecule compounds. First, we showed that IP-FCM can detect statistically significant differences in samples possessing a defined PPI change as low as 10%. This sensitivity allowed IP-FCM to detect a PPI that increases transiently during T cell signaling, the antigen-inducible interaction between ZAP70 and the T cell antigen receptor (TCR)/CD3 complex. In contrast, IP-FCM detected no ZAP70 recruitment when T cells were stimulated with antigen in the presence of the src-family kinase inhibitor, PP2. Further, we tested whether IP-FCM possessed sufficient sensitivity to detect the effect of a second, rare class of compounds called SMIPPI (small-molecule inhibitor of PPI). We found that the first-generation non-optimized SMIPPI, Ro-26-4550, inhibited the IL-2:CD25 interaction detected by IP-FCM. This inhibition was detectable using either a recombinant CD25-Fc chimera or physiologic full-length CD25 captured from T cell lysates. Thus, we demonstrate that IP-FCM is a sensitive tool for measuring physiologic PPIs that are modulated by signal transduction and pharmacologic inhibition.

## Introduction

Cell signaling pathways often involve numerous protein-protein interactions (PPI) in processes as diverse as receptor:ligand binding, signal transduction across physical barriers, and translocation of signals between different cellular compartments [1], [2]. Together, these PPIs are thought to form a system with emergent network properties, integrating signals from a variety of inputs into coordinated responses. By this means, PPIs play central roles in cellular growth, and many other processes associated with either healthy or diseased states [3], [4], [5]. Currently, there is significant interest in the generation of biotechnological assays that would display sufficient sensitivity to detect subtle changes in PPIs, changes of a magnitude on scale with those that occur in response to distinct physiologic conditions. Ideally, such high-sensitivity PPI assays could also be useful in drug development, if they could be proved capable of detecting the effects on PPI that result from drug targeting.

Pharmacologically, some PPIs can be indirectly targeted if drugs alter the activity of upstream enzymes or other regulatory processes. However, PPIs have long been considered difficult direct drug targets for small organic molecules [6]. Because the surface area of a PPI interface is relatively long and flat, involving the summation of many minor interactions, PPI disruption is expected to occur only rarely due to the binding of a single small drug [7]. Nevertheless, recent studies have demonstrated that, while rare, small-molecule inhibitors of PPI (SMIPPI) can be found. They work by binding to “hot spots”, regions of the interaction interface that contribute significantly more to the binding energy of the PPI than do other regions [8]. Part of the attraction of SMIPPIs is that they are theorized to display the long-sought attribute that has so often failed in the search for kinase inhibitors: high specificity. The prediction is that SMIPPI might provide increased on-target specificity and fewer side effects than drugs targeting enzymes; by targeting only a specific interaction of a given pair of proteins, those proteins might still perform other non-pathologic functions. Leading compounds for the few SMIPPI reported to date were identified by various strategies, but most involved random screening of chemical libraries [9], [10], [11]. The pace of drug development for PPI inhibitors could be accelerated by the development of rapid, inexpensive assays with high sensitivity and robustness, capable of screening the enormous libraries of potentially bioactive compounds now available.

**Figure 1:**
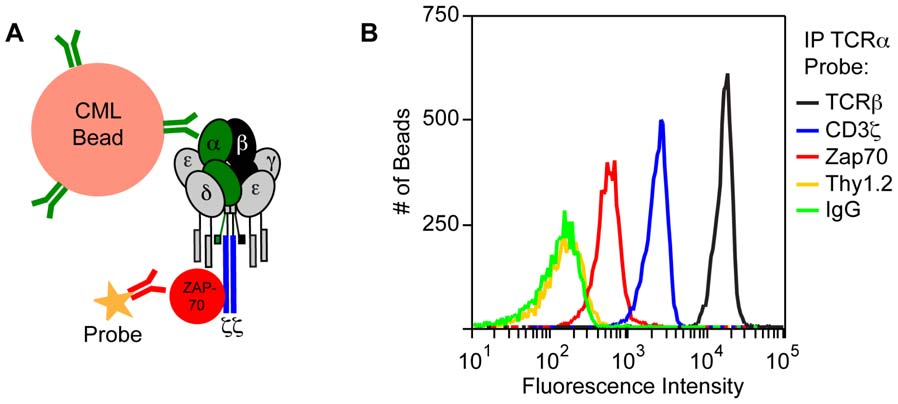
IP-FCM detects physiologic PPIs associated with the TCR/CD3 complex. (**A**) IP antibodies to TCRα (green) are covalently coupled to CML microspheres, which are incubated with T cell lysates to immunoprecipitate the multiprotein complex (TCR/CD3 subunits plus ZAP70, as indicated). Fluorescently labeled probe antibodies specific for different components of the complex (in this case Zap70, red), can be used to detect the association between the primary target and associated proteins. (**B**) Anti-TCRα-conjugated CML beads were used to immunoprecipitate TCR/CD3 complexes from Tot1.1 cells. These complexes were negative when probed with irrelevant IgG (green), or with anti-Thy1.2 (yellow), the latter being specific for a highly expressed membrane protein that is not part of the complex. However, TCRα was present in shared complexes with TCRβ (black) and CD3ζ (blue). ZAP70 (red) also showed a lower but measurable association.

We have previously described a method of measuring the PPIs of stable protein complexes based on immunoprecipitation followed by flow cytometry (IP-FCM) [12], [13], [14], [15], [16]. In IP-FCM, carboxylate-modified polystyrene latex microspheres (CML beads) are covalently coupled to antibodies specific to a given target protein. Fluorochrome-conjugated probe antibodies can then detect either the immunoprecipitated target in a sandwich ELISA-style assay, or co-immunoprecipitated proteins bound to the immunoprecipitated target (Figure 1A). Analysis of the beads by flow cytometry produces semi-quantitative fluorescence intensity data over a broad reportable range. IP-FCM can assess physiologic proteins in their native state, and does not require artificial over-expression of proteins or expression in non-mammalian hosts. The technique is amenable to analysis of transmembrane proteins, which are important components of signal transduction pathways but can be difficult to analyze in other microassay formats with high-throughput capability.

The aim of the present study was to determine the compatibility of IP-FCM with assessment of PPIs that are modulated during signal transduction and/or pharmacologic inhibition. We report that IP-FCM is able to measure the transient, inducible increase in ZAP70 association with the TCR/CD3 complex upon physiologic TCR engagement, an event which can be blocked by the small molecule src-family kinase inhibitor, PP2. Additionally, we test the sensitivity of IP-FCM using a first-generation SMIPPI, Ro-26-4550, which interferes with the binding of IL-2 to CD25 (IL-2 receptor, α-subunit) [17]. These studies demonstrate that IP-FCM displays high sensitivity to small changes in physiologic PPI, whether induced by signal transduction or inhibited by small-molecule compounds. We posit that IP-FCM represents a technological strategy that could make a significant contribution to large scale screening for potential pharmacologic modulators of PPI.

## Materials and Methods

### Antibodies

The following antibodies were used: Anti IL-2: MQ1-17H2 (capture reagent, Biolegend), poly5111 (probe reagent, Biolegend); Anti-CD25: 7G7 (capture reagent, Upstate Biotech, now EMD Millipore), 24024 (capture reagent, R&D Systems), 24212 (probe reagent, R&D systems); Anti-TCRα (Vα2-specific): B20.1 (capture reagent, isolated from hybridoma supernatant); Anti-TCRβ: H57 (probe reagent, BD Biosciences or Biolegend); Anti-CD3ζ: 6B10 (probe reagent, Santa Cruz Biotech); Anti ZAP70: 1E7 (probe reagent, BD Biosciences or eBioscience); Anti-Thy1.2: 30-H12 (probe reagent, BD Biosciences or Biolegend). All capture antibodies were used as purified immunoglobulins in Phosphate Buffered Saline (PBS) at the time of coupling to microspheres, as described below. Probe antibodies were either purchased in conjugated form to fluorescein isothiocyanate (FITC) or R-phycoerythrin (PE), or were biotinylated as described below, and detected using the secondary reagent streptavidin (SA) conjugated to either FITC or PE.

### Preparation of Microspheres and Probes

Capture and probe antibodies were either purchased in PBS or exchanged into PBS as described below. Amicon Ultra spin filters with 10 kDa MWCO were used to concentrate antibodies and to buffer-exchange to remove primary amine-containing buffers such as Tris. Antibodies purchased in buffers containing Bovine Serum Albumin (BSA) were purified three times using the MelonGel IgG Spin Purification Kit (Thermo Scientific, USA). Conjugation to microspheres was performed as previously described [14]. Briefly, CML latex microspheres (Invitrogen #C37255, USA) were activated with EDAC (1-ethyl-3-(3-dimethylaminopropyl) carbodiimide HCl; Pierce, USA), and coupled to 50 µl of 0.5 mg/ml antibody for 3 hours at room temperature. Unlabeled probe antibodies were biotinylated at 0.5 mg/ml with EZ-link Sulfo-NHS-Biotin (Thermo, USA); excess unreacted biotin was removed by buffer exchange using spin filters.

### IP-FCM of the TCR/CD3 Complex

Tot 1.1 cells [12] were grown in RPMI with 10% Cosmic Calf serum (HyClone, Thermo Scientific), and were subsequently centrifuged, supernatant discarded, and cell pellets frozen at −20°C until use. For most experiments, frozen cell pellets were lysed at 2×10^8^ (abbreviated scientific notation, 2e^8^) cells per ml in lysis buffer (150 mM NaCl, 50 mM Tris, 1% Digitonin, Halt protease/phosphatase inhibitor (Pierce)). For the cell lysate dilution series, cells were lysed at 5e^8^ cells per ml and were diluted two-fold in lysis buffer 15 times, three replicates per data point. For the 10% change assay, ten replicates of cells were diluted to 6.25e^7^, 5.625e^7^ or 5e^7^ cells per ml. Immunoprecipitation (IP) was performed by adding 2.5e^4^ antibody-conjugated beads to 10 µl of cell lysate per IP. After overnight incubation at 4°C, biotinylated probe antibodies were added for 2 hours on ice, followed by washing and 1 hour incubation with SA-PE. Alternatively, PE-conjugated antibodies were added for 2 hours on ice. CML beads were then analyzed on a flow cytometer (either Accuri C6, BD FACScan, or BD FACSCalibur). The beads were gated using forward and side scatter, and the PE fluorescence of each bead was measured. Median PE values and histograms of PE-height were used for analysis.

### IP-Western Blotting

Tot1.1 cells were lysed as described above, and two-fold dilutions were prepared. Cell lysates (1 ml) were incubated overnight at 4°C with 1 µg anti-TCR Vα2 antibody (B20.1), followed by IP for 2 hours with 20 µl protein G magnetic beads (New England Biolabs, Inc.). Beads were magnetically separated, washed twice, and boiled for five minutes in reducing SDS buffer. Samples were run on a 13% SDS-PAGE gel and transferred to nitrocellulose membranes. Blots were probed with rabbit monoclonal anti-ZAP70 (clone 99F2, R&D systems), and anti-rabbit-IgG-Horse Radish Peroxidase (HRP) secondary antibody (Santa Cruz Biotechnology). Luminol reagent was used as substrate (Santa Cruz Biotechnology), and luminescence was captured on photographic film (Kodak), which was subsequently scanned on an Epson 10000X scanner.

### IP-FCM of the CD25:IL-2 Complex

Human peripheral blood mononuclear cells (PBMC) were Ficol-purified from leukoreduction system chambers (LRSC) as described [18] in accordance with the Mayo Clinic College of Medicine’s Institutional Review Board (IRB) regulations. Cells were stimulated overnight with 4 ng/ml phorbol myristate acetate (PMA) and 1 µM Ionomycin to induce CD25 expression before freezing of cell pellets at −20°C until use. Either 20 µl of cell lysate (1e^8^ cells/ml in lysis buffer) or 10 µl of recombinant human CD25-Fc chimera (R&D Systems, 37.5 µg/ml) was incubated with 2.5e^4^ anti-CD25 beads overnight. Beads were washed and recombinant human IL-2, (Proleukin®, Prometheus Labs, USA) was added for 1 hour, with or without the addition of 1 or 10 µM Ro-26-4550 (Tocris, USA) or control compounds. Beads were washed and then incubated with anti-IL2 or anti-CD25 probe antibodies and analyzed as described above.

### Stimulation of OT1 T Cells

Mouse whole splenocytes and lymphocytes were harvested from OT1 transgenic mice after euthanasia by carbon dioxide asphyxiation in accordance with the Mayo Clinic College of Medicine’s Institutional Animal Care and Use Committee (IACUC) regulations. T2Kb cells were grown in complete IMEM supplemented with 10% FCS and 400 µg/ml G418. T2Kb cells were loaded with peptides by incubation with 2 µM peptide in IMEM for two hours, then briefly fixed in 0.05% glutaraldehyde and washed several times in PBS, as we have described previously [19], [20], [21]. The H2-K^b^ restricted peptides FARL (SSIEFARL), Q4H7 (SIIQFEHL) and OVA (SIINFEKL) were purchased from Elim Biopharmaceuticals, Inc. In experiments involving the src-family kinase inhibitor PP2 (Sigma, USA), OT1 splenocytes and lymphocytes were pre-incubated with 10 µm PP2 or control compounds for one hour. OT1 cells were then mixed with T2Kb cells on ice at a ratio of 0.7 T2Kb/OT1. Cells were pelleted at 4°C, the supernatant removed, and pellets were incubated at 37°C for various times before addition of lysis buffer, incubation on ice for 20 minutes, and clarification of the lysate by centrifugation. IP and analysis proceeded as described above.

### Statistics

**Figure 2:**
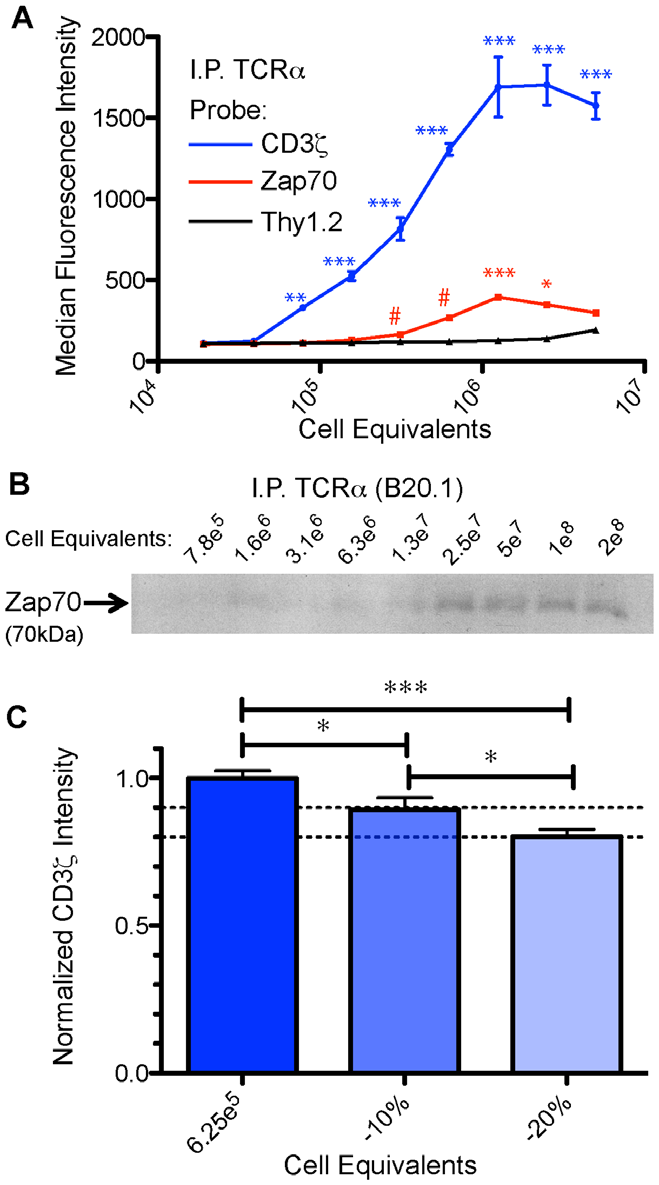
IP-FCM detects PPIs over a wide range of protein concentrations, with high sensitivity. (**A**) Nine serial 2-fold dilutions of a Tot1.1 cell lysate were immunoprecipitated with anti-TCRα-coupled CML beads and probed with anti-CD3ζ, anti-ZAP70, or anti-Thy1.2 antibodies in triplicate. The TCR:CD3 interaction was significantly detectable over background (i.e., over Thy1.2 nonspecific signal) at concentrations above 7.8e^6^ cells per ml (2-way ANOVA followed by post-hoc comparisons of CD3 vs Thy1.2; **p<0.01, ***p<0.001). ZAP70 association was detectable over background at concentrations above 3.1e^7^ cells per ml (# indicates data points that are significant when Student’s t-test is used to compare each data point to the corresponding Thy1.2 background data point, see Results). When applying multiple comparison corrections, detection of ZAP70 association was statistically significant at 1.25e^8^ cells per ml (2-way ANOVA followed by post-hoc comparison of ZAP70 vs Thy1.2, *p<0.05, ***p<0.001). (**B**) A series of nine 2-fold dilutions of Tot1.1 cell lysate was subjected to IP using anti-TCR Vα2 antibody and protein G magnetic beads. Captured complexes were subjected to SDS-PAGE and Western blot for ZAP70. (**C**) To assess IP-FCM sensitivity, ten replicates of Tot1.1 cell lysate (6.25e^7^ cells per ml) were diluted by 10% and 20%, immunoprecipitated for TCRα and the captured complexes probed for CD3ζ. One-way ANOVA followed by post-hoc testing revealed a significant difference between all dilutions (*p<0.05, ***p<0.001).

**Figure 3:**
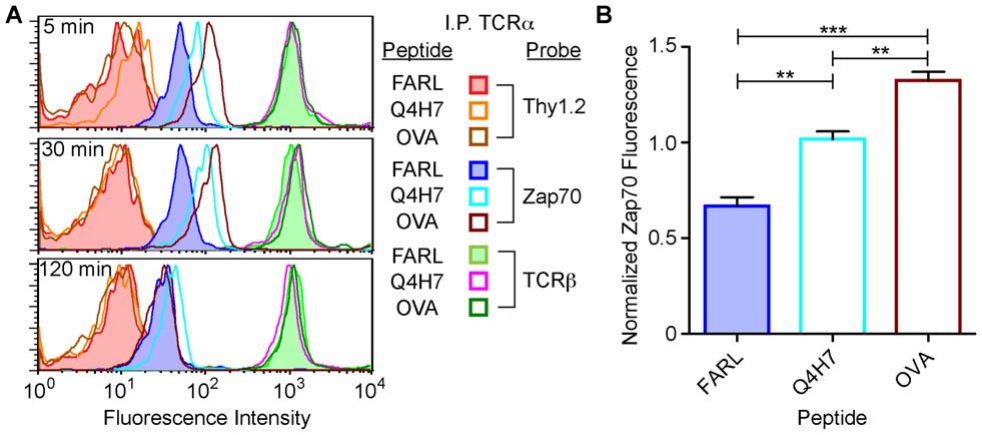
IP-FCM detects antigen-induced transient increase in ZAP70 association with TCR/CD3. OT1 TCR transgenic T cells were co-incubated with peptide-loaded T2Kb APCs for up to two hours. The APCs had been pulsed with either FARL or OVA peptides presented in H-2K^b^. Following stimulation, cells were lysed and subjected to IP-FCM analysis. (**A**) ZAP70 association with TCR/CD3 increased in OVA- and Q4H7-stimulated cells (OVA: dark brown, open histogram; Q4H7: aqua, open histogram), when compared with FARL (blue, filled histograms). This difference was maintained by 30 minutes, but disappeared by 120 minutes. (**B**) For the ZAP70 recruitment data in each of 3 independent experiments, normalized values were calculated as described in Materials and Methods in order to assess the difference in fluorescence of each experimental group from the mean (set to 1) of all three experimental groups. This normalization manipulation allowed the data from the 3 independent experiments to be considered on a single scale while subjected together to one-way ANOVA followed by Newman-Keuls post-hoc tests. A significant difference was observed between OVA and FARL, OVA and Q4H7, and Q4H7 and FARL-induced ZAP70 association with TCR/CD3 (ANOVA: F_2,9_ = 55.8, p<0.0001; **p<0.01, ***p<0.005).

**Figure 4:**
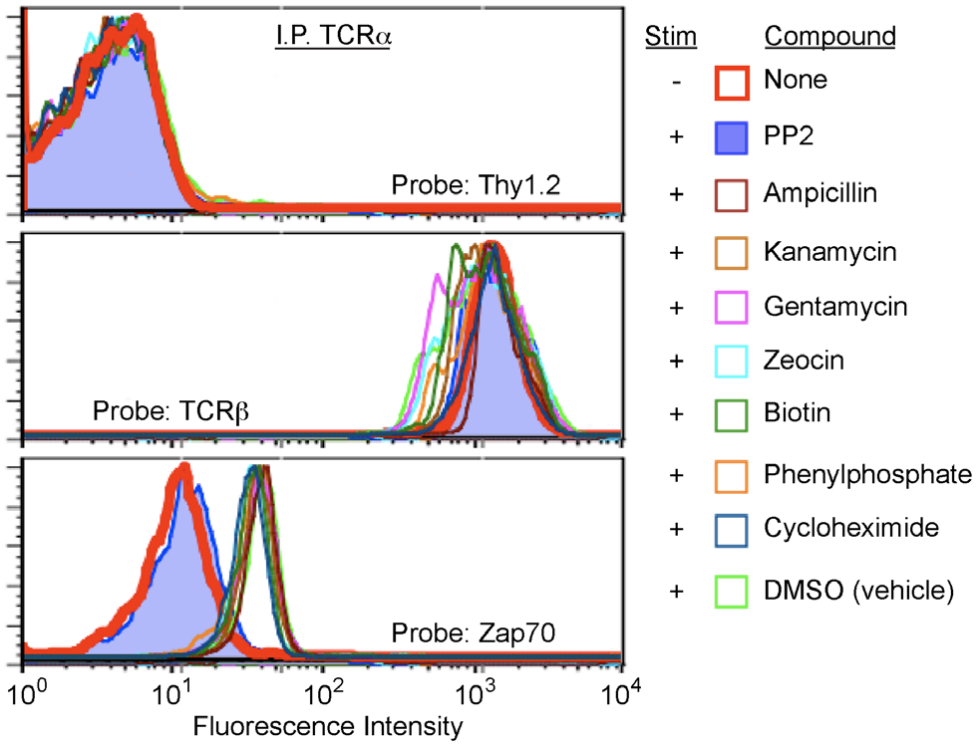
IP-FCM detects the specific inhibition of antigen-inducible ZAP70 recruitment by the small molecule src-family kinase inhibitor, PP2. OT1 TCR transgenic T cells were pre-incubated with 10 µM of either PP2, a known src-kinase inhibitor, or one of several other small-molecule control compounds. After one hour, T cells were co-incubated for 5 minutes with T2Kb APCs presenting OVA. Following incubation, T cells were lysed and subjected to IP-FCM analysis. Only in the presence of PP2 did IP-FCM detect inhibition of ZAP70 recruitment to TCR/CD3.

**Figure 5:**
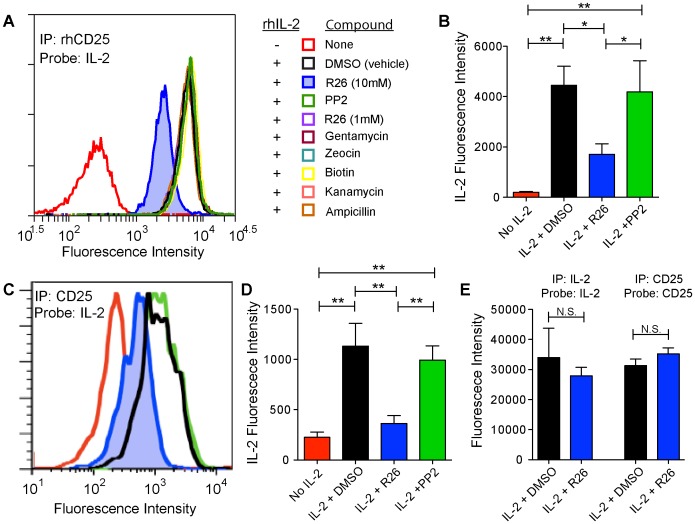
IP-FCM detects the specific inhibition of CD25:IL-2 interaction by the first-generation SMIPPI, Ro-26-4550. (**A**) Recombinant human CD25 (rhCD25) was immunoprecipitated with anti-CD25 antibody-conjugated CML beads. Recombinant human IL-2 (1000 U/ml) and one of several small-molecules were mixed with the beads for one hour, followed by probing with an anti-IL-2 antibody. Inhibition of the IL2:CD25 interaction was observed only upon addition of 10 mM R0-26-4550 (R26, blue shaded histogram). (**B**) Median fluorescence intensity from three experiments shows R26 significantly reduced IL-2:CD25 binding compared to either vehicle (DMSO) or PP2 negative controls (ANOVA: F_3,10_ = 10.82, p = 0.0051; **p<0.01, *p,0.05) (**C**) Endogenous CD25 was immunoprecipitated from lysates from PMA/Ionomycin-stimulated human PBMCs, and analyzed as in (A) using 10,000 U/ml IL-2. (**D**) Median fluorescence intensity from four experiments including (C) shows R26 significantly reduced IL-2:CD25 binding compared to either vehicle (DMSO) or PP2 negative controls (ANOVA: F_3,15_ = 10.07, p = 0.0013; **p<0.01) (**E**) Experiments for (C–D) included IL-2 and CD25 ELISAs that were performed. Compared to negative controls, R26 did not change the amount of IL2 or CD25 detected (N.S, not significant by Student’s T-test, p>>0.05), indicating that the difference in (C–D) was due altered IL-2:CD25 interaction.

Scientific notation of format A x 10^b^ is abbreviated in this article as Ae^b^. Statistics were analyzed using Prism (Graphpad) software. In the lysate dilution experiments (Figure 2A), 2-way ANOVA followed by Bonferroni post-hoc tests were used to compare the averaged median fluorescence intensities. In the 10% change experiments (Figure 2B), 1-way ANOVA followed by Newman-Keuls post-hoc tests were used to compare averaged median florescence values. Power analysis was performed using StatMate (Graphpad) software by either inputting measured standard deviations and Ns to estimate the power of the experiment as performed, or by inputting the average of experimentally observed standard deviations with Ns of 2 (duplicate measurements) or 3 (triplicate measurements) to estimate the power of the IP-FCM assay. In the ZAP70 recruitment experiments (Figures 3–4), 1-way ANOVA followed by Newman-Keuls post-hoc tests were used to compare normalized median florescence values. Normalized values were calculated as follows: Normalized ZAP70_Peptide_  =  {ZAP70_Peptide_/{(ZAP70_FARL_+ZAP70_Q4H7_+ZAP70_OVA_)/3}}. Effectively, this normalization method allowed measurement of the difference of each data point from the mean (set to 1). This manipulation minimized the differences in raw fluorescence between experiments, thus allowing the data from independent experiments to be used together for statistical testing. In the SMIPPI experiments (Figure 5), 1-way ANOVA followed by Newman-Keuls post-hoc tests or Student’s t-test was used to compare median fluorescence values. All data are plotted as mean +/− SEM.

### Ethics Statement

Mice were housed, euthanized, and their tissues used for experiments in accordance with Mayo Clinic’s Institutional Animal Care and Use Committee (IACUC) regulations (AAALAC accreditation number 000717; USDA registration number 41-R-0006; OLAW assurance number A3291-01).

## Results

### IP-FCM for the Transmembrane TCR/CD3 Multiprotein Complex and ZAP70

Our model multiprotein complex is αβ T cell antigen receptor (TCR)/CD3: the TCR proper is composed of a disulfide-linked αβ heterodimer, while CD3 is composed of subunits γ, δ, ε, and ζ (Figure 1; Reference [22]). At a low basal level which increases upon TCR engagement, the src-family kinases Lck or Fyn phosphorylate specific immunoreceptor tyrosine-based activation motifs (ITAM) on CD3 cytoplasmic tails, creating binding sites for the Syk-family kinase ZAP70 [23]. To analyze these complexes, TCRα was immunoprecipitated from lysate of unstimulated Tot1.1 cells, which represent a tissue culture-passaged thymic leukemia cell line that spontaneously arose in an OT1 TCR-transgenic mouse in our colony [12]. Captured protein complexes were probed for either TCRβ, CD3ζ, ZAP70 or Thy1.2 (Figure 1B). The TCRβ and CD3ζ probes demonstrated inclusion of these proteins in shared complexes with TCRα, as anticipated. The ZAP70 probe displayed a low positive signal, matching the expectation that a basal amount was associated with antigen-unstimulated TCR/CD3 complexes [24]. The Thy1.2 probe acts as a control for membrane disassociation; since Thy1.2 is not part of the TCR/CD3 complex, the level if any of Thy1.2 observed with TCRα defines the background association level in the absence of PPI [12], [15]. We conclude that IP-FCM can detect TCR/CD3 and ZAP70 in shared complexes.

To assess assay sensitivity and determine the statistical power of IP-FCM under these conditions, cell lysate dilution experiments were performed. Serial two-fold dilutions were made across a wide range of lysate cell concentrations (5e^8^ to 3e^4^ cells/ml), immunoprecipitated for TCRα and probed for CD3ζ, ZAP70, and Thy1.2 as a negative control (Figure 2A). The TCR:CD3 association was detectable from as little as 7.8e^6^ cell equivalents per ml (2-way ANOVA with probe and concentration of lysate as independent variables: F_14,89_ = 133.5; F_2,89_ = 677.1 respectively, p<0.0001; Bonferroni post-hoc testing showed p<0.001 for cell concentrations greater than 7.8e^6^ cells/ml). Detection of ZAP70 association with TCR/CD3 was significantly above background at 3.1e^7^ or 6.2e^7^ cells/ml, if each concentration was treated as independent and subjected to Student’s t-test (as would be the case in standard experiments performed at a single concentration). By two-way ANOVA post-hoc testing for multiple comparison corrections, detection of ZAP70 association was significant at 1.25e^8^ cells/ml (Figure 2A). For comparison, robust visualization of the association required a greater amount of cellular material when a Tot1.1 lysate dilution range was subjected to IP for TCR followed by Western blotting for ZAP70 (Figure 2B).

We next tested the ability of IP-FCM to detect a 10% or 20% change within the detectable range by performing ten replicates of the TCR/CD3 co-IP assay at concentrations of 6.25e^7^, 5.625e^7^ (less 10%) or 5e^7^ (less 20%) cell equivalents per ml. Median fluorescence values were normalized to the average median fluorescence of the most concentrated data point (Figure 2C). The means +/− standard deviations of the ten normalized median fluorescence values were 1+/−0.079, 0.89+/−0.128 and 0.80+/−0.077. One-way ANOVA followed by Newman-Keuls post-hoc tests indicated a significant difference (p<0.05) between the 10% data points, and a significant difference (p<0.001) between the 20% data points (ANOVA: F_2,29_ = 10.34, p<0.0005). Power analysis using data from the sets of ten replicates revealed 99% power to detect a change in assay signal as low as 14%, with 70% –85% power to detect a change of 10%. Since IP-FCM is usually performed in duplicate or triplicate, we then averaged the standard deviations of the three dilution sets to approximate an assay-wide standard deviation, and asked what percent change the assay had the power to detect (calculations not shown). This analysis revealed 99% power to detect a 50% change (if duplicates are used) or a 36% change (if triplicates are used), and 80% power to detect a 32% change (duplicates) or a 23% change (triplicates). We conclude that IP-FCM shows high sensitivity to detect relatively small changes in PPI levels.

### Antigen-inducible, Transient Increase in ZAP70 Association with TCR/CD3

We sought to determine whether IP-FCM could distinguish unstimulated and stimulated levels of ZAP70 association with TCR/CD3. Naïve, primary OT1 TCR-transgenic T cells were stimulated with antigen-presenting cells (APCs) that had been pulsed with a negative control peptide (FARL), an antagonist peptide (Q4H7), or the nominal antigenic peptide (OVA), a system we have used previously [19], [20], [21]. After incubation times of 5, 30 or 120 minutes, T cells were lysed and subjected to IP-FCM using IP-beads specific for the OT1 transgenic TCR α-chain, and probe antibodies targeted to TCRβ, ZAP70 or Thy1.2. We observed that ZAP70 association with TCR/CD3 was influenced by both the peptide and the time of incubation (Figure 3). Stimulation with APCs presenting the antigenic peptide (OVA) or the antagonist peptide (Q4H7) induced a rapid increase in the amount of complex-associated ZAP70 when compared to the inactive peptide, FARL, although the agonist induced greater recruitment than the antagonist (Figure 3A–B). ZAP70 association remained elevated at 30 minutes, but disappeared by two hours post-stimulation. Throughout the time course, the TCR:Thy1.2 measurement remained low, indicating successful membrane dissociation and unaltered assay background. Furthermore, the association of TCR αβ throughout the experiment was high and served as a “loading control”, showing that similar numbers of complexes were captured on all IP-beads. We conclude that IP-FCM is capable of detecting increased levels of ZAP70 recruitment in response to a physiologically relevant signaling event, the engagement and activation of the TCR by antigen presentation.

### IP-FCM Detects Drug-induced Inhibition of ZAP70 Recruitment to TCR/CD3

In order to determine the ability of IP-FCM to detect drug-induced changes in PPI, we treated primary OT1 T cells with the src-family kinase inhibitor PP2 prior to antigenic stimulation. PP2 inhibits Lck and Fyn activity in T cells, thus preventing the phosphorylation of CD3 and the recruitment of ZAP70 to the TCR/CD3 complex. As specificity controls, OT1 cells were treated in parallel with seven other small-molecule control compounds. Stimulation with OVA-loaded APCs increased ZAP70 association with TCR/CD3 compared to FARL-loaded APCs (Figure 4). However, treatment with PP2 inhibited OVA-induced ZAP70 recruitment, which did not increase above basal (FARL) levels (Figure 4). Neither the seven pathway-irrelevant control compounds nor the vehicle control (DMSO) affected ZAP70 recruitment. Importantly, the constitutive TCRα/β interaction remained high regardless of stimulatory condition or the small-molecule treatments, while the Thy1.2 control remained at unaltered background levels. These data demonstrate that IP-FCM is able to detect specific drug-induced inhibition of a physiologic PPI.

### IP-FCM Detects Inhibition of IL-2:CD25 Interaction by a First-generation SMIPPI

Ro-26-4550 (R26) is a first generation SMIPPI that interferes with the binding of IL-2 and CD25 (IL-2 receptor, α-chain) [17]. R26 competitively inhibits the IL2-CD25 interaction with an IC_50_ of 3 µM [25]. Although subsequently optimized derivatives of R26 show improved target affinity [26], we chose to use the first generation molecule to best mimic a weaker compound of the type that might be identified in an initial drug screen. We first captured recombinant human CD25 onto beads coated with an anti-CD25 antibody. Beads were then incubated with recombinant human IL-2 (1,000 U/ml) for one hour, along with R26 or various non-specific small-molecule or vehicle controls. Beads were then washed and probed with anti-IL-2 antibodies. A no-compound, no-IL-2 condition defined the background level of the assay. Under these conditions, we observed that 10 mM R26 reduced the interaction of CD25 with IL2 by 62% (Figure 5A,B). Neither 1 mM R26 nor the seven small-molecule controls inhibited the PPI, demonstrating both the specificity and the R26-concentration-dependence of inhibition. Similar results were obtained by adding 500 U/ml IL-2 (56% reduction of IL-2:CD25 binding) and 10,000 U/ml IL-2 (44% reduction), demonstrating the detection of inhibition over a range of analyte concentrations (data not shown).

Because many PPI partners, such as transmembrane domain-containing proteins, are not easily engineered in a full-length form that is also soluble in a recombinant system, we used stimulated, primary human PBMCs as a source of full-length, endogenous CD25. PBMC cell lysates were immunoprecipitated overnight with anti-CD25 beads, and the beads were incubated with 10,000 U/ml IL-2 alone or in combination with 10 µM R26, or alternatively PP2, in this case a negative control compound. Again, significant inhibition of CD25:IL-2 interaction was observed in the presence of R26 (Figure 5C,D). Importantly, R26 did not inhibit the ELISA-style detection of either IL-2 or CD25, indicating that R26 specifically inhibited the CD25:IL2 interaction without affecting capture and detection of either partner separately (Figure 5E). Therefore, using either recombinant material or material derived from primary physiologic sources, IP-FCM detected the inhibition of a PPI by a first-generation SMIPPI.

## Discussion

There is significant interest in the generation of microassays that could be applied to PPI analysis [27]. Desirable attributes of such microassays include compatibility with high-throughput formatting and potential for assessing PPI signatures or phenotypes from small biomedical samples such as those commonly obtained in the clinic. Furthermore, microassays with such a high level of sensitivity could potentially be utilized in drug screening strategies in search of leading compounds that alter PPI by indirect or direct mechanisms. We have previously reported that IP-FCM possesses several of these desirable properties when assessing stable PPI from small samples [14]. In the current work, we show that IP-FCM displays high sensitivity to small changes in physiologic PPI, and can be applied to transient PPI that are modulated by signal transduction and/or pharmacologic interference. These attributes make IP-FCM attractive as a method that could assess physiologic PPI signatures, or could be applied to drug screening.

We observed that IP-FCM detected the interaction between TCR and CD3 over a wide range of protein concentrations. Power analysis revealed that when samples are assayed in duplicate, the IP-FCM shows a 99% chance of detecting a 50% change, and 80% chance of detecting a 32% change; when performed in triplicate the assay has a 99% chance of detecting a 36% change and an 80% chance of detecting a 23% change in protein concentration. The wide range of detection coupled with high sensitivity to detect small changes in PPIs makes IP-FCM attractive for applications including physiologic PPI phenotyping or discovery of leading compounds during small-molecule drug screening.

IP-FCM detected the low baseline interaction between ZAP70 and TCR/CD3 over an approximately three-fold concentration range. Furthermore, upon antigenic stimulation of primary T cells, IP-FCM clearly detected significant ZAP70 recruitment. It is well established in the literature that upon engagement of TCR by its physiologic ligand, peptide/MHC, the src-family kinases Lck or Fyn rapidly phosphorylate ITAMs on the cytoplasmic tails of the CD3 subunits, creating binding sites for ZAP70. This initiates a signaling cascade which leads to T cell activation in a coordinated cellular response [23]. In our experiments, inducible ZAP70 recruitment peaked at 5 minutes and disappeared by two hours, showing this to be a transient, signal-transduction-dependent event. IP-FCM also detected the blockade of inducible ZAP70 recruitment by the src-family kinase inhibitor PP2, demonstrating the assay’s sensitivity to PPI modulation by pharmacological interference.

We next assessed the extent to which IP-FCM might detect SMIPPI activity. SMIPPIs offer great promise as a new drug class that could disrupt pathogenic PPIs with high specificity, such that unwanted side effects might be minimized [28]. We found that IP-FCM detected the effect of the first-generation SMIPPI Ro-26-4550 (R26) in both fully recombinant and partially native systems. We detected a strong (∼50%) inhibition of CD25:IL2 interaction over a ∼20-fold range of IL-2 concentrations. As a first-generation SMIPPI, more potent forms of R26 have been developed since its initial characterization [17]. However, we intentionally used the earliest version of the compound to mimic the type of assay that would be performed in an early discovery screen. Under these conditions, IP-FCM detected PPI inhibition in a simple ex-vivo assay, over a broad range of target concentrations, and using a SMIPPI concentration compatible with high-throughput screening. Thus, we feel that IP-FCM displays significant potential as a method that could be applied to screening for drugs with SMIPPI activity.

We envision IP-FCM as a unique tool with many applications. Besides the potential utility in drug screening discussed above, IP-FCM offers several advantages over traditional IP-Western blotting, including reduced protein input requirements (Figure 2A–B), increased speed, potential to convert semi-quantitative to quantitative data [14], [15], and high reproducibility. On the other hand, IP-FCM does not provide molecular weight information, but instead restricts data to protein identification and quantification, as is typical of ELISA-style methods. Required equipment includes a flow cytometer capable of analyzing CML beads, in which case the Accuri C6 is not much more expensive than a digital gel dock for Western blotting. Further, as a microsphere-based method, IP-FCM offers the potential for multiplex formatting, as we have recently reported [12].

In conclusion, IP-FCM can assess physiologic PPIs over a wide range of protein concentrations using a small amount of biological starting material. We demonstrate that the assay can detect changes in PPI as low as 10%. We further show that IP-FCM can detect the low basal interaction of ZAP70 with TCR/CD3, its increase upon physiologic stimulation, and the inhibition of its recruitment by the src-kinase inhibitor, PP2. Finally, we show that IP-FCM detected the inhibition of the PPI between CD25 and IL2 caused by the first-generation SMIPPI, Ro-26-4550. We propose that IP-FCM represents a sensitive method for PPI analysis with high potential to make a significant contribution to physiologic PPI profiling in response to signal transduction and pharmacologic modulation.
